# Prognostic value of longitudinal strain relative apical sparing in severe aortic stenosis patients undergoing TAVR

**DOI:** 10.1002/ehf2.15365

**Published:** 2025-07-27

**Authors:** Dan Liu, Kai Hu, Vera Schimpf, Victoria Sokalski, Friederike Hermann, Björn Daniel Lengenfelder, Georg Ertl, Stefan Frantz, Peter Nordbeck

**Affiliations:** ^1^ Department of Internal Medicine I University Hospital Würzburg Würzburg Germany; ^2^ Comprehensive Heart Failure Center Würzburg Germany

**Keywords:** Heart failure, Hypoalbuminemia, Longitudinal strain, Relative apical sparing, Transcatheter aortic valve replacement

## Abstract

**Aims:**

This study evaluated the prognostic value of the relative apical sparing pattern (RASP) of longitudinal strain (LS) in patients with severe aortic stenosis (AS) undergoing transcatheter aortic valve replacement (TAVR) and investigated whether its combination with pre‐procedural biomarkers enhances risk stratification.

**Methods and results:**

This retrospective study included 598 patients (mean age 81.7 ± 5.7 years, 48.8% male) with severe AS undergoing TAVR. Two‐dimensional speckle‐tracking echocardiography was used to assess LS. RASP was defined as an apical‐to‐basal LS ratio >3.0 in ≥3 out of six left ventricular walls. The primary endpoint was 2‐year cardiovascular (CV) mortality. RASP was present in 19.2% of patients and independently predicted 2‐year CV mortality (hazard ratio [HR] 2.01, 95% CI 1.22–3.29, *P* = 0.006). Low serum albumin (<4.0 g/dL; HR 2.40, 95% CI 1.50–3.84, *P* < 0.001) and low BMI (≤25.5 kg/m^2^; HR 1.71, 95% CI 1.07–2.73, *P* = 0.025) were also independent predictors. A composite risk score (0–3 points) was constructed using these three factors. Two‐year CV mortality increased progressively with higher scores: 6.3% for score 0, 11.4% for score 1, 27.2% for score 2 and 35.3% for score 3 (log‐rank *P* < 0.001). High‐risk patients (score ≥2) had a more than threefold increase in adjusted mortality risk (HR 3.42, 95% CI 2.14–5.48, *P* < 0.001).

**Conclusions:**

RASP, particularly when combined with hypoalbuminemia and low BMI, identifies a high‐risk phenotype associated with adverse outcomes after TAVR. This integrated risk model may assist in guiding pre‐procedural assessment and individualized management.

## Introduction

Recent studies have identified the relative apical sparing pattern (RASP) of longitudinal strain (LS) as a significant echocardiographic marker, characterized by preserved apical LS relative to basal and mid‐segments.^1^ Traditionally associated with cardiac amyloidosis (CA),^2^, ^3^ RASP is increasingly recognized in severe aortic stenosis (AS) patients,^4^, ^5^, ^6^ even in the absence of confirmed amyloid infiltration.^7^ Research has shown that RASP is not only prevalent among AS patients but also linked to distinct echocardiographic features, including adverse left ventricular (LV) remodelling and impaired diastolic function. Notably, RASP has been associated with worse cardiovascular outcomes, such as higher mortality rates, potentially reflecting underlying myocardial alterations beyond amyloidosis.^8^, ^9^

Transcatheter aortic valve replacement (TAVR) has emerged as a cornerstone treatment for severe AS, particularly in elderly patients with high surgical risk. Despite its benefits, post‐TAVR outcomes remain heterogeneous, underscoring the need for improved risk stratification tools. Recent studies have suggested a relationship between RASP and increased all‐cause mortality in severe AS patients undergoing TAVR,^10^ yet the independent prognostic value of RASP in this setting remains insufficiently explored.

Therefore, this study aimed to evaluate the prognostic significance of RASP in predicting cardiovascular outcomes in severe AS patients following TAVR. Additionally, we sought to determine whether integrating RASP with other clinical and echocardiographic factors, including pre‐procedural biomarkers, could enhance risk stratification and better identify high‐risk patient subgroups.

## Methods

### Study population

This retrospective cohort study comprised 598 consecutive symptomatic severe AS patients referred for TAVR in our hospital between July 2009 and October 2018. All patients were elected for a TAVR intervention after discussion by a heart team. The European System for Cardiac Operative Risk Evaluation (EuroSCORE) II models were used to predict risk of in‐hospital mortality after cardiac surgery. Patients underwent TAVR using the balloon expandable Edwards‐Sapien bioprosthesis (Edwards Sapien/Sapien XT, Edwards Lifesciences, Irvine, California) through the transfemoral (*n* = 397, 66.4%) or transapical (*n* = 201, 33.6%) route. The study conformed to the principles outlined in the Declaration of Helsinki and was approved by the Local Ethics Committee at the University of Wuerzburg. Written informed consent was obtained from all patients or their guardians prior to study start.

### Standard echocardiography assessment

Standard transthoracic echocardiographic examination was performed before procedure (GE, Vingmed Vivid 7 or IE9, Horten, Norway). Measurements were made offline according to the current guidelines in a remote workstation (EchoPAC version 113, GE, Horten, Norway).^11^, ^12^ LV end‐diastolic dimension (LVEDD), end‐diastolic thickness of the posterior wall (LVPWd) and the septum (IVSd) were measured using M‐mode in the parasternal LV long‐axis view. Left ventricular ejection fraction (LVEF) was measured with the biplane Simpson method in apical 4‐ and 2‐chamber views. LV mass indexed to body surface area (LVMi), was estimated by LV cavity dimension and wall thickness at end diastole: LV mass (g) = 0.8 × [1.04 × (LVEDD + LVPWd + IVSd)^3^ – LVEDD^3^)] + 0.6. Relative wall thickness (RWT) was calculated using the following formula: 2 × LVPWd/LVEDD. A LV concentric hypertrophy was defined as LVMi >95 g/m^2^ for women or >115 g/m^2^ for men and RWT > 0.42.^13^ Diastolic dysfunction (DD) was graded according to the current recommendations in 2016.^14^ Peak velocity of early (E) and atrial (A) diastolic filling and deceleration time of E wave (DT) were measured as well as the E/A ratio was calculated. Tissue‐Doppler derived early diastolic mitral annular velocity (E′) was acquired at the septal mitral annular sites and then septal E/E′ were calculated.

### Longitudinal strain and bull's eye plots

Two‐dimensional speckle tracking imaging analysis was performed using EchoPAC software (GE, Horten, Norway), and the LS bull's eye plots were acquired according to the procedure described previously.^1^, ^15^ In brief, the bull's eye plot was obtained with the standard 2D strain method. A region of interest is created by manually applying successive points along the endocardial border in the three apical views (i.e., apical 4 chamber, 2 chamber and long‐axis views) at end‐systolic frame, respectively. The LV is divided into six walls and three segments in each LV wall, accounting for 18 segments covering the entire LV from base to apex. LS of each segment from LV six walls and global LS (GLS) derived from 18 segments were measured.

### Definitions of RASP

**Figure 1:**
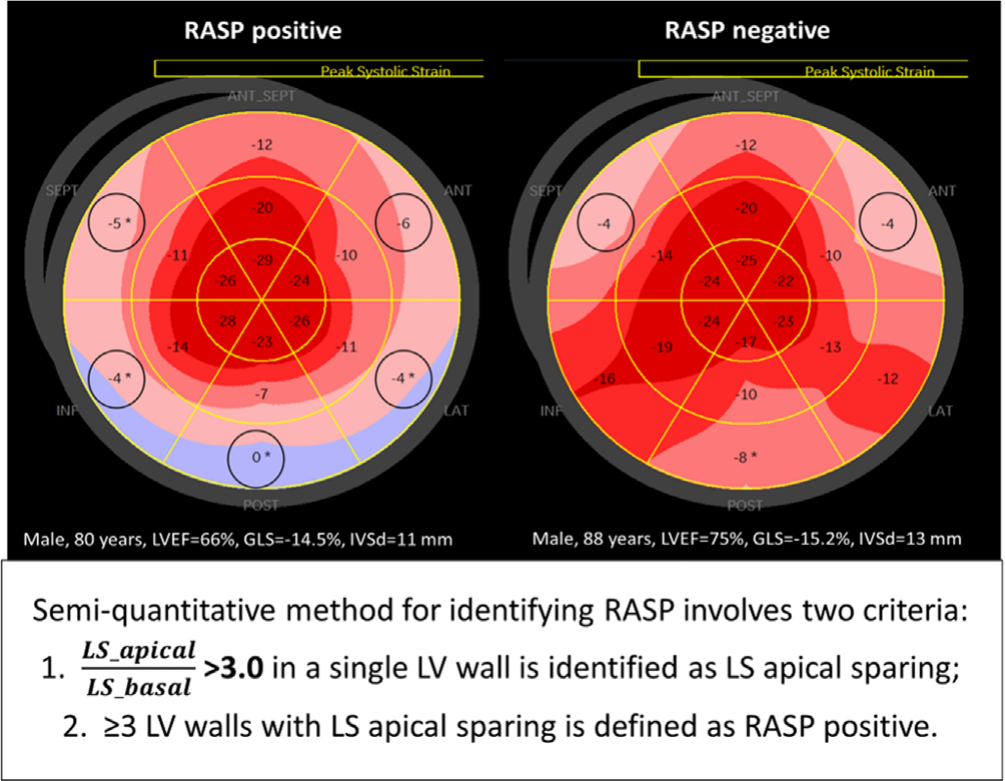
Definition and examples of the relative apical sparing pattern (RASP) of left ventricular longitudinal strain (LS) in severe aortic stenosis patients underwent TAVR. Semiquantitative method for identifying RASP involves two criteria: (1) LS_apical/LS_basal >3.0 in a single LV wall is identified as LS apical sparing; (2) ≥ 3 LV walls with LS apical sparing is defined as RASP positive. The left panel displays a 18‐segment LS bull's eye plot with a RASP positive in a patient underwent TAVR (80 years, male, LVEF 66%, GLS −14.5%), and the right panel displays a 18‐segment LS bull's eye plot with a RASP negative in a TAVR patient (88 years, male, LVEF 75%, GLS −15.2%). GLS, global longitudinal strain of left ventricle; LVEF, left ventricular ejection fraction; TAVR, transcatheter aortic valve replacement.

RASP was assessed using both quantitative and semiquantitative methods, in accordance with established methodologies described in previous studies.^3^ The quantitative method defined RASP as an apical‐to‐basal GLS ratio, calculated as $\frac{\mathrm{GLS}\_\text{apical}}{\mathrm{GLS}\_\mathrm{mid}+\mathrm{GLS}\_\text{basal}}$ >1.0. In addition to the quantitative method, we introduced a novel semiquantitative approach to define RASP (*Figure* *1*). For each LV wall, the apical‐to‐basal LS ratio (LS_apical/LS_basal) was calculated. Apical LS sparing was defined as an apical‐to‐basal LS ratio >3.0 in any given LV wall. The total number of LV walls exhibiting apical LS sparing was then counted to determine the presence of RASP. Given that the optimal threshold for the number of LV walls with apical LS sparing associated with clinical outcomes was previously unknown, we conducted diagnostic testing to identify the most predictive cut‐off point. This included an evaluation of sensitivity, specificity, positive predictive value (PPV) and negative predictive value (NPV).

### Outcomes

Enrolled patients completed a follow‐up of up to 2‐year through consulting medical records or telephone interviews. Clinical outcomes were determined based on the updated endpoint definitions for aortic valve clinical research by The Valve Academic Research Consortium 3 (VARC‐3).^16^ Primary endpoints was defined as cardiovascular (CV) death from any cause at 2 years post TAVR. CV deaths were defined as deaths from an acute myocardial infarction, sudden cardiac death, death due to heart failure, death due to stroke, death due to CV procedures, death due to CV haemorrhage, death due to other CV causes, or heart transplantation. Death from unknown causes were assumed to be deaths from CV causes. Procedure related and other CV related hospitalization and neurologic events (ischaemic or haemorrhagic stroke) at 30 days, 1 year and 2 years post TAVR were also documented. Follow‐up data at 30 days focused on mortality, neurologic events, CV prehospitalization, bleeding and transfusions, minor or major vascular and assess‐related complications, procedural or valve related complications (such as aortic root rupture, coronary occlusion, etc.), acute kidney injury (AKI), new‐onset left bundle branch block (LBBB) and new permanent pacemaker implantations. AKI was defined as a no less than 150% increase in serum creatinine (SCr) within 7 days post TAVR or a 0.3 mg/dL increase in absolute SCr value within 48 h post TAVR.

### Statistical analysis

Statistical analyses were performed using SPSS 26.0 (SPSS Inc., Chicago, IL, USA). All statistical tests were two‐tailed, and a *P* value <0.05 was considered statistically significant. Continuous variables were presented as mean ± standard deviation or median (interquartile range, IQR). Differences between two groups were compared using unpaired Student's *t* test or Mann–Whitney *U* test, as appropriate. Categorical variables were expressed as count and per cent, and the differences between groups were compared using chi‐square test or Fischer's exact test, as indicated.

Multivariable binary logistic regression was performed to identify independent factors associated with the presence of RASP, and variables exhibiting a significant difference between groups (*P* < 0.05) entered into the model. Odd ratios (ORs) with 95% confidence interval (CI) were calculated. Survival curves were estimated using the Kaplan–Meier method and compared by the log‐rank test. Multivariable binary logistic regression models were also used to identify risk factors associated with AKI and new‐onset LBBB within 30 days post TAVR. Multivariable Cox proportional hazard regression models were used to determine risk factors associated with 1‐year and 2‐year CV mortality. Adjusted hazard ratios (HRs) with 95% CI were calculated. Prognostic parameters were incorporated into either binary logistic models or Cox regression models using the ‘Enter’ method. The median values of key prognostic parameters in patients who experienced CV death within 2 years were initially used as cutoff points for survival analysis. Receiver operating characteristic (ROC) curve analysis was performed to determine the optimal cutoff value of absolute global longitudinal strain (GLS_Avg) for predicting the presence of RASP, and of body mass index (BMI) for predicting 2‐year CV mortality. The Youden index was applied to identify optimal thresholds. Potential collinearity among variables was assessed using Spearman's rho correlation test. Variables demonstrating a Spearman's rho greater than 0.40 were identified as indicative of collinearity. In such cases, only one of the collinear variables was included in the model to reduce the effects of multicollinearity.

## Results

### Pre‐procedural clinical and echocardiographic features

The cohort consisted of 598 patients with a mean age of 81.7 ± 5.7 years, of whom 48.8% were male (*Table* *1*). The median EuroSCORE II was 5.0% (IQR: 3.2–8.7%). The median clinical follow‐up period was 24 months (IQR: 17–24 months). The 2‐year CV mortality rate was 12.4% (74 out of 598). Patients who experienced CV death within 2 years after TAVR had significantly higher proportions of BMI ≤ 25.5 kg/m^2^ (52.7% vs. 35.9%, *P* = 0.005) and EuroSCORE II > 6.9% (50.0% vs. 34.4%, *P* = 0.009) compared with survivors. Additionally, the CV death group showed significantly lower levels of haemoglobin (median: 11.7 vs. 12.4 g/dL, *P* < 0.001) and serum albumin (median: 4.1 vs. 4.3 g/dL, *P* < 0.001).

**Table 1:** Pre‐procedural clinical and echocardiographic characteristics associated with 2‐year cardiovascular (CV) mortality post‐TAVR

	Total	No CV‐death	CV‐death	*P* value
No.	598 (100)	524 (87.6)	74 (12.4)	
Age (years)	81.7 ± 5.7	81.7 ± 5.7	81.8 ± 5.8	0.893
Male [*n* (%)]	291 (48.7)	253 (48.3)	38 (51.4)	0.621
Body mass index (kg/m^2^)	27.1 ± 4.5	27.2 ± 4.5	26.3 ± 5.1	0.103
≤25.5 kg/m^2^	227 (38.0)	188 (35.9)	39 (52.7)	0.005
Systolic blood pressure (mmHg)	135.6 ± 24.3	136.3 ± 24.5	130.8 ± 23.0	0.097
Diastolic blood pressure (mmHg)	70.6 ± 13.7	70.6 ± 13.8	70.9 ± 13.4	0.865
EuroSCORE II (%)	5.0 (3.2–8.7)	5.0 (3.1–8.3)	6.9 (4.4–10.8)	0.006
>6.9%	217 (36.3)	180 (34.4)	37 (50.0)	0.009
NYHA class II/III/IV (%)	15.1/73.4/9.5	15.3/74.0/8.6	13.5/68.9/16.2	0.212
Comorbidities (%)				
Obesity	22.9	23.5	18.9	0.383
Atrial fibrillation	41.6	40.3	51.4	0.070
Hypertension	81.4	81.3	82.4	0.814
Diabetes	33.8	34.0	32.4	0.794
Dyslipidaemia	64.0	64.7	59.5	0.380
Hyperuricemia	10.2	10.1	10.8	0.853
Coronary artery disease	56.4	56.1	58.1	0.745
Previous myocardial infarction	13.2	13.5	10.8	0.515
Previous PCI	30.3	30.3	29.7	0.914
Previous CABG	12.2	12.2	12.2	0.990
Stroke or transient ischaemic attack	17.1	16.8	18.9	0.649
Peripheral vascular disease	11.9	11.1	17.6	0.106
Chronic respiratory diseases	23.1	22.1	29.7	0.147
Chronic renal dysfunction	58.9	58.6	60.8	0.716
Permanent pacemaker	9.7	9.7	9.5	0.941
Biochemical parameters				
Creatinine (mg/dL)	1.19 (0.95–1.50)	1.18 (0.95–1.47)	1.29 (0.93–1.68)	0.188
eGFR (mL/min/1.73 qm)	55.5 (41.0–70.0)	56.0 (41.3–70.0)	48.0 (36.8–73.3)	0.230
Haemoglobin (g/dL)	12.3 (11.2–13.3)	12.4 (11.2–13.4)	11.7 (10.8–12.7)	<0.001
Albumin (g/dL)	4.2 (4.0–4.4)	4.3 (4.1–4.5)	4.1 (3.8–4.3)	<0.001
TAVR approach				0.016
Transfemoral	397 (66.4)	357 (68.1)	40 (54.1)*	
Transapical	201 (33.6)	167 (31.9)	34 (45.9)*	
Standard echocardiography measurements				
LVEF (%)	57.0 ± 13.0	56.9 ± 13.2	57.5 ± 11.1	0.726
≥50%	453 (75.8)	397 (75.8)	56 (75.7)	0.987
<50%	145 (24.2)	127 (24.2)	18 (24.3)	
LVEDD (mm)	47.9 ± 7.6	48.0 ± 7.6	47.1 ± 7.7	0.320
IVSd (mm)	11.7 ± 2.0	11.8 ± 2.0	11.7 ± 2.1	0.814
LVPWd (mm)	11.2 ± 1.9	11.2 ± 1.8	11.0 ± 2.1	0.402
LVMi (g/m^2^)	114.4 ± 32.2	114.9 ± 32.2	110.8 ± 32.2	0.305
RWT	0.48 ± 0.13	0.48 ± 0.13	0.49 ± 0.15	0.891
LV concentric hypertrophy (%)	37.0	37.6	32.4	0.389
LAVi (mL/m^2^)	44.8 ± 18.9	44.2 ± 18.7	49.3 ± 19.8	0.030
RAA (cm^2^)	17.9 ± 6.3	17.7 ± 6.1	19.4 ± 7.0	0.034
RVD_mid (mm)	27.8 ± 6.7	27.8 ± 6.6	28.3 ± 7.2	0.555
TAPSE (mm)	18.5 ± 4.9	18.6 ± 4.9	17.3 ± 4.5	0.030
MAPSE_septal (mm)	7.9 ± 2.1	8.0 ± 2.1	7.5 ± 1.9	0.043
MAPSE_lateral (mm)	9.3 ± 2.3	9.3 ± 2.4	9.1 ± 2.1	0.458
sPAP (mmHg)	41.1 ± 13.6	40.4 ± 13.2	45.7 ± 15.4	0.002
Septal E/E′	23.4 ± 10.6	22.8 ± 10.1	27.4 ± 12.2	0.002
DD grade (%)				0.094
Mild	37.5	38.7	28.4	
Moderate	50.0	49.6	52.7	
Severe	12.5	11.6	18.9	
AVV_max_ (m/s)	4.2 ± 0.7	4.2 ± 0.7	4.1 ± 0.6	0.176
AVP_mean_ (mmHg)	46.9 ± 16.2	47.4 ± 16.4	43.5 ± 14.0	0.056
AVAi (cm^2^/m^2^)	0.44 ± 0.11	0.44 ± 0.11	0.44 ± 0.10	0.931
SVi (mL/m^2^)	43.5 ± 10.5	43.7 ± 10.4	42.2 ± 10.6	0.249
Moderate to severe AR (%)	14.0	14.5	10.8	0.392
Moderate to severe MR (%)	22.7	23.1	20.3	0.588
Moderate to severe MAC (%)	13.2	13.0	14.9	0.653
2D speckle tracking derived longitudinal strain				
18‐segment GLS_Avg	−14.1 ± 3.9	−14.2 ± 4.0	−14.1 ± 3.6	0.944
Absolute value >15.5%	232 (38.8)	208 (39.7)	24 (32.4)	
Absolute value ≤15.5%	366 (61.2)	316 (60.3)	50 (67.6)	0.230
GLS_apical	−19.1 ± 6.1	−19.1 ± 6.2	−19.6 ± 5.6	0.472
GLS_mid	−13.0 ± 3.8	−13.0 ± 3.9	−13.0 ± 3.6	0.999
GLS_basal	−10.3 ± 3.4	−10.4 ± 3.4	−9.9 ± 3.4	0.245
Apical‐basal GLS ratio^a^	0.85 ± 0.25	0.84 ± 0.25	0.89 ± 0.26	0.092
RASP definition				
If defined as apical‐basal GLS ratio > 1.0^a^	128 (21.4)	108 (20.6)	20 (27.0)	0.208
If defined as ≥3 out of six LV walls with apical‐basal LS ratio > 3.0^b^	115 (19.2)	89 (17.0)	26 (35.1)	<0.001

^a^ Apical‐basal GLS ratio = $\frac{\mathrm{GLS}\_\text{apical}}{\mathrm{GLS}\_\mathrm{mid}+\mathrm{GLS}\_\text{basal}}$.

^b^ Apical‐basal LS ratio of each LV wall = $\frac{\mathrm{LS}\_\text{apical}}{\mathrm{LS}\_\text{basal}}$.

AR, aortic regurgitation; AVAi, aortic valve area indexed to body surface area; AVP_mean_, mean transaortic gradient; AVV_max_, maximum transaortic velocity; CABG, coronary artery bypass grafting; DD, diastolic dysfunction; E/E′, ratio of early transmitral Doppler flow velocity to early diastolic tissue velocity (septal); eGFR, estimated glomerular filtration rate; EuroSCORE, European System for Cardiac Operative Risk Evaluation; GLS_apical, GLS averaged by six apical segments; GLS_Avg, global longitudinal strain averaged by 18 segments; GLS_basal, GLS averaged by six basal segments; GLS_mid, GLS averaged by six mid segments; IVSd, end‐diastolic wall thickness of the septum; LAVi, end‐systolic left atrial volume indexed to body surface area; LS_apical, apical longitudinal strain of one LV wall; LS_basal, basal longitudinal strain of one LV wall;LV, left ventricular; LVEDD, end‐diastolic left ventricular dimension; LVEF, left ventricular ejection fraction; LVMi, left ventricular mass indexed to body surface area; LVPWd, end‐diastolic wall thickness of the left ventricular posterior wall; MAC, mitral annular calcification; MAPSE, mitral annular plane systolic excursion; MR, mitral regurgitation; NYHA, New York Heart Association; PCI, percutaneous coronary intervention; RAA, end‐systolic right atrial area; RVD_mid, end‐diastolic right ventricular mid diameter; RWT, relative wall thickness; sPAP, systolic pulmonary artery pressure; SVi, stroke volume indexed to body surface area; TAPSE, tricuspid annular plane systolic excursion; TAVR, transcatheter aortic valve replacement. .

In terms of echocardiographic parameters, patients with CV death had a higher left atrial volume index (LAVi: 49.3 ± 19.8 vs. 44.2 ± 18.7 mL/m^2^, *P* = 0.030) and right atrial area (RAA: 19.4 ± 7.0 vs. 17.7 ± 6.1 cm^2^, *P* = 0.034). They also presented with reduced tricuspid annular plane systolic excursion (TAPSE) and mitral annular plane systolic excursion (MAPSE) (*P* < 0.05 for both), along with higher septal E/E′ ratios (27.4 ± 12.2 vs. 22.8 ± 10.1, *P* = 0.002) and elevated systolic pulmonary artery pressure (sPAP: 45.7 ± 15.4 vs. 40.4 ± 13.2 mmHg, *P* = 0.002). The average 18‐segment GLS for the entire cohort was −14.1 ± 3.9%. There was no significant difference in GLS between the CV‐death and no CV‐death groups (−14.1 ± 3.6% vs. −14.2 ± 4.0%, *P* = 0.944), suggesting that GLS was not correlated with the risk of CV death post‐TAVR.

*Table* S1 summarizes the clinical and echocardiographic parameters significantly associated with 30‐day and 1‐year CV mortality, including BMI, haemoglobin, albumin, septal E/E′ and sPAP (*P* < 0.05 for all). In multivariable Cox regression analyses (Table S2), lower BMI, reduced serum albumin and elevated septal E/E′ emerged as independent predictors of increased 1‐ and 2‐year CV mortality after TAVR, after adjustment for relevant clinical covariates.

### Prevalence of RASP

The prevalence of quantitative RASP was 21.4% in the entire cohort. There were no significant differences in the prevalence of quantitative RASP between patients with and without CV death at 30 days (21.4% vs. 21.7%, *P* = 1.000), 1 year (20.8% vs. 26.8%, *P* = 0.302), or 2 years (20.6% vs. 27.0%, *P* = 0.208) post‐TAVR. To establish the optimal threshold for semiquantitative RASP, we performed diagnostic testing, evaluating sensitivity, specificity, PPV and NPV for 2‐year CV mortality (Table S3). The presence of apical LS sparing in ≥3 out of six LV walls demonstrated the strongest diagnostic performance, with a sensitivity of 35.2%, specificity of 82.9%, PPV of 21.7%, and NPV of 90.5%. Based on these findings, semiquantitative RASP was defined as the presence of apical LS sparing in ≥3 LV walls and was applied for subsequent analyses in this study. The prevalence of semiquantitative RASP was 19.2% in the entire cohort, and it was significantly higher in the CV‐death group compared with the no CV‐death group: 30 days post‐TAVR (34.8% vs. 18.6%, *P* = 0.062), 1 year post‐TAVR (32.1% vs. 17.9%, *P* = 0.010) and 2 years post‐TAVR (35.1% vs. 17.0%, *P* < 0.001).

### Independent determinants associated with semiquantitative RASP

Patients exhibiting semiquantitative RASP had a significantly higher EuroSCORE II (median 5.8% vs. 5.0%, *P* = 0.043), a greater proportion with BMI ≤ 25.5 kg/m^2^, lower prevalence of obesity and higher prevalence of atrial fibrillation and hyperuricemia. Additionally, they showed lower serum albumin levels compared with those without RASP (*Table* *2*). Furthermore, RASP was associated with LV concentric hypertrophy, increased LAVi and RAA, elevated septal E/E′ and sPAP, as well as reduced TAPSE and MAPSE. The prevalence of moderate to severe DD was higher among RASP patients. Moderate to severe mitral annular calcification (MAC) was also more common in the RASP group compared with the no‐RASP group (20.9% vs. 11.4%, *P* = 0.007). In terms of myocardial deformation, patients with RASP had significantly lower absolute GLS values (−12.8 ± 3.4% vs. −14.5 ± 3.9%, *P* < 0.001) and a higher prevalence of absolute GLS ≤ 15.5% (82.6% vs. 56.1%, *P* < 0.001).

**Table 2:** Pre‐procedural clinical and echocardiographic characteristics in TAVR patients with and without RASP and independent determinants associated with the presence of RASP

	No RASP	RASP	*P* value	Univariable OR (95% CI)	*P* value	Multivariable OR (95% CI)^a^	*P* value
No. (%)	483 (80.8)	115 (19.2)					
Age (years)	81.6 ± 5.5	82.2 ± 6.3	0.276				
Male [*n* (%)]	235 (48.7)	56 (48.7)	0.994				
Body mass index (kg/m^2^)	27.2 ± 4.6	26.4 ± 4.0	0.070				
≤25.5 kg/m^2^	174 (36.0)	53 (46.1)	0.046	1.518 (1.006–2.290)	0.047		
Systolic blood pressure (mmHg)	135.8 ± 23.7	134.7 ± 26.6	0.692				
Diastolic blood pressure (mmHg)	70.2 ± 13.1	72.1 ± 16.0	0.246				
EuroSCORE II (%)	5.0 (3.0–8.5)	5.8 (3.9–9.5)	0.043	1.037 (1.001–1.074)	0.042		
>6.9%	168 (34.8)	49 (42.6)	0.117				
NYHA class >2 [*n* (%)]	397 (82.2)	93 (80.9)	0.740				
Comorbidities [*n* (%)]							
Obesity	119 (24.6)	18 (15.7)	0.039	0.568 (0.329–0.978)	0.041		
Atrial fibrillation	189 (39.1)	60 (52.2)	0.011	1.697 (1.127–2.554)	0.011		
Hypertension	391 (81.0)	96 (83.5)	0.531				
Diabetes	164 (34.0)	38 (33.0)	0.853				
Dyslipidaemia	311 (64.4)	72 (62.6)	0.721				
Hyperuricemia	42 (8.7)	19 (16.5)	0.013	2.078 (1.158–3.731)	0.014	2.027 (1.049–3.916)	0.036
Coronary artery disease	269 (55.7)	68 (59.1)	0.504				
Previous myocardial infarction	63 (13.0)	16 (13.9)	0.805				
Previous PCI	140 (29.0)	41 (35.7)	0.162				
Previous CABG	64 (13.3)	9 (7.8)	0.110				
Stroke or transient ischaemic attack	84 (17.4)	18 (15.7)	0.656				
Peripheral vascular disease	52 (10.8)	19 (16.5)	0.086				
Chronic respiratory diseases	116 (24.0)	22 (19.1)	0.264				
Chronic renal dysfunction	276 (57.1)	76 (66.1)	0.080				
Permanent pacemaker	52 (10.8)	6 (5.2)	0.071				
Biochemical parameters							
Creatinine (mg/dL)	1.18 (0.95–1.47)	1.23 (0.96–1.62)	0.472				
eGFR (mL/min/1.73 qm)	56.0 (41.0–70.0)	51.0 (41.0–72.0)	0.331				
Haemoglobin (g/dL)	12.3 (11.1–13.3)	12.4 (11.4–13.6)	0.273				
Albumin (g/dL)	4.3 (4.0–4.5)	4.1 (3.9–4.4)	0.010	0.624 (0.372–1.045)	0.073		
Standard echocardiography							
LVEF (%)	57.4 ± 13.1	55.3 ± 12.3	0.110				
≥50%	369 (76.4)	84 (73.0)					
<50%	114 (23.6)	31 (27.0)	0.451				
LVEDD (mm)	48.1 ± 7.6	47.0 ± 7.7	0.116				
IVSd (mm)	11.6 ± 2.0	12.1 ± 1.9	0.015				
LVPWd (mm)	11.1 ± 1.8	11.5 ± 2.0	0.049				
LVMi (g/m^2^)	114.0 ± 32.7	116.0 ± 30.0	0.582				
RWT	0.48 ± 0.13	0.51 ± 0.14	0.019				
RWT > 0.42	305 (63.1)	85 (73.9)	0.029				
LV concentric hypertrophy	166 (34.4)	55 (47.8)	0.007	1.751 (1.160–2.641)	0.008	1.605 (1.018–2.532)	0.042
LAVi (mL/m^2^)	43.7 ± 17.8	49.5 ± 22.1	0.006	1.015 (1.004–1.025)	0.005		
RAA (cm^2^)	17.7 ± 6.2	19.0 ± 6.4	0.050	1.033 (1.002–1.066)	0.038		
RVD_mid (mm)	27.9 ± 6.7	27.4 ± 6.6	0.435				
TAPSE (mm)	18.7 ± 4.8	17.3 ± 5.0	0.002	0.939 (0.900–0.981)	0.004		
MAPSE_septal (mm)	8.1 ± 2.1	7.3 ± 1.8	<0.001	0.815 (0.733–0.907)	<0.001		
MAPSE_lateral (mm)	9.5 ± 2.3	8.4 ± 2.1	<0.001	0.805 (0.731–0.886)	<0.001		
sPAP (mmHg)	40.3 ± 13.4	44.1 ± 13.7	0.009	1.020 (1.005–1.036)	0.009		
Septal E/E′	22.6 ± 10.4	26.5 ± 10.7	<0.001	1.032 (1.014–1.051)	<0.001		
Moderate to severe DD	286 (59.2)	88 (76.5)	<0.001	2.245 (1.406–3.584)	<0.001	1.958 (1.099–3.486)	0.023
AVV_max_ (m/s)	4.2 ± 0.7	4.2 ± 0.7	0.646				
AVP_mean_ (mmHg)	47.0 ± 15.8	46.5 ± 17.7	0.690				
AVAi (cm^2^/m^2^)	0.44 ± 0.11	0.41 ± 0.09	0.004	0.047 (0.006–0.374)	0.004		
SVi (mL/m^2^)	44.3 ± 10.4	40.3 ± 10.2	<0.001	0.962 (0.942–0.982)	<0.001		
Moderate to severe AR	72 (14.9)	12 (10.4)	0.215				
Moderate to severe MR	106 (21.9)	30 (26.1)	0.341				
Moderate to severe MAC	55 (11.4)	24 (20.9)	0.007	2.052 (1.208–3.487)	0.008	2.091 (1.139–3.838)	0.017
18‐segment GLS_Avg	−14.5 ± 3.9	−12.8 ± 3.4	<0.001				
Absolute value ≤15.5%	271 (56.1)	95 (82.6)	<0.001	3.716 (2.221–6.216)	<0.001	3.280 (1.870–5.755)	<0.001

^a^ Multivariable binary logistic regression was performed, incorporating variables that demonstrated a significant inter‐group difference (*P* < 0.05) using the ‘Enter’ method. A Spearman's rho correlation of 0.420 was observed between septal E/E′ and moderate to severe DD, and a correlation of 0.410 between MAPSE_septal and GLS_Avg. To address potential multicollinearity, septal E/E′ and MAPSE_septal were excluded from the multivariable binary regression model.

AR, aortic regurgitation; AVAi, aortic valve area indexed to body surface area; AVP_mean_, mean transaortic gradient; AVV_max_, maximum transaortic velocity; CABG, coronary artery bypass grafting; CI, confidence interval; DD, diastolic dysfunction; E/E′, ratio of early transmitral Doppler flow velocity to early diastolic tissue velocity (septal); eGFR, estimated glomerular filtration rate; EuroSCORE, European System for Cardiac Operative Risk Evaluation; GLS_Avg, global longitudinal strain averaged by 18 segments; IVSd, end‐diastolic wall thickness of the septum; LAVi, end‐systolic left atrial volume indexed to body surface area; LV, left ventricular; LVEDD, end‐diastolic left ventricular dimension; LVEF, left ventricular ejection fraction; LVMi, left ventricular mass indexed to body surface area; LVPWd, end‐diastolic wall thickness of the left ventricular posterior wall; MAC, mitral annular calcification; MAPSE, mitral annular plane systolic excursion; MR, mitral regurgitation; NYHA, New York Heart Association; OR, odds ratio; PCI, percutaneous coronary intervention; RAA, end‐systolic right atrial area; RASP, relative apical sparing pattern of longitudinal strain, defined as more than three out of six LV walls with an apical‐to‐basal LS ratio >3.0; RVD_mid, end‐diastolic right ventricular mid diameter; RWT, relative wall thickness; sPAP, systolic pulmonary artery pressure; SVi, stroke volume indexed to body surface area; TAPSE, tricuspid annular plane systolic excursion; TAVR, transcatheter aortic valve replacement.

In multivariable binary logistic regression analysis, the following variables were independently associated with the presence of RASP: hyperuricemia (OR = 2.027, *P* = 0.036), LV concentric hypertrophy (OR = 1.605, *P* = 0.042), moderate to severe DD (OR = 1.958, *P* = 0.023), moderate to severe MAC (OR = 2.091, *P* = 0.017) and absolute average GLS ≤ 15.5% (OR = 3.280, *P* < 0.001).

### Clinical outcomes associated with RASP at 30 days, 1 year and 2 years post TAVR

**Figure 2:**
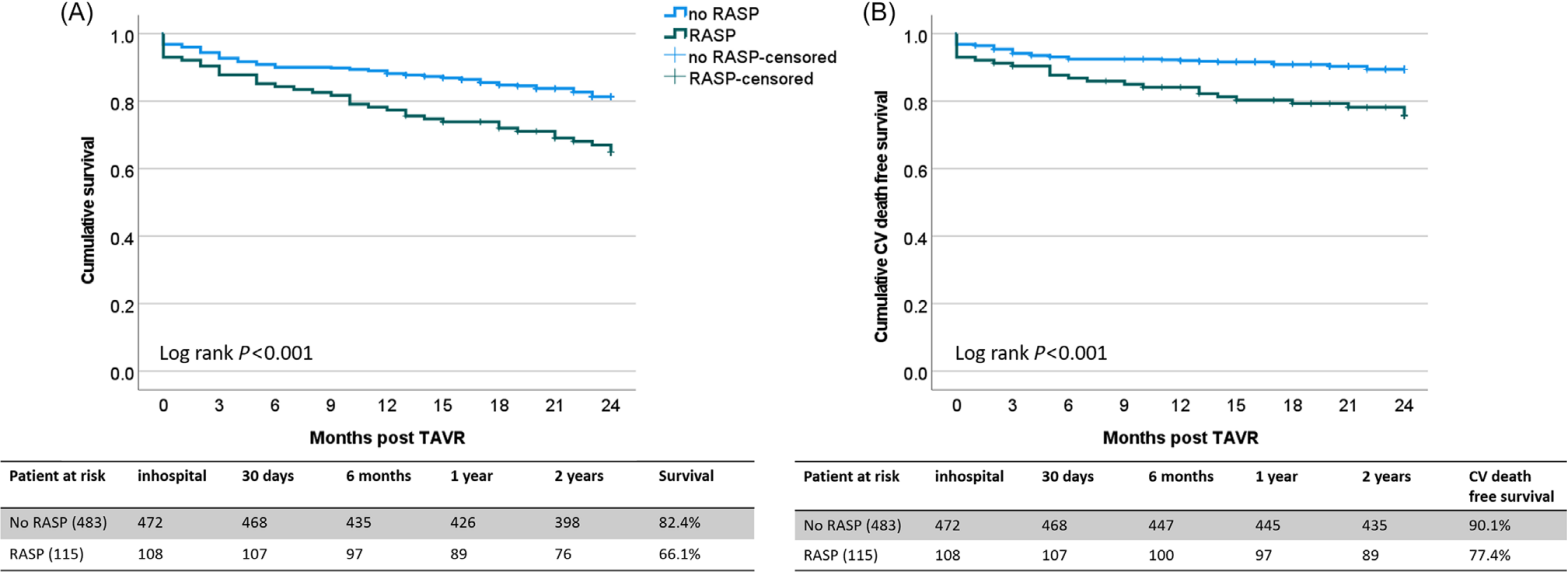
Kaplan–Meier curves of the relative apical sparing pattern (RASP) of left ventricular longitudinal strain for predicting 2‐year overall survival (A) and CV events‐free survival (B) in severe aortic stenosis patients underwent TAVR. CV, cardiovascular; TAVR, transcatheter aortic valve replacement.

At 30 days post‐TAVR, the CV mortality was 3.8%, which tended to be higher in the RASP group compared with the non‐RASP group (7.0% vs. 3.1%, *P* = 0.062). The prevalence of AKI (10.4% vs. 3.3%, *P* = 0.001) and new onset LBBB post TAVR (17.4% vs. 6.8%, *P* < 0.001) were significantly higher in the RASP group than that in the non‐RASP group. Neurologic events (1.5%), bleeding and transfusions (1.3%), vascular and assess‐related complications (1.8%), procedural or valve‐related complications (1.3%), and new permanent pacemaker implantation post‐TAVR were similar between the two groups. At both 1‐year and 2‐year follow‐ups post‐TAVR, all‐cause mortality and CV mortality were significantly higher in the RASP group compared with the non‐RASP group (*Table* *3* and *Figure* *2* *A,B*).

**Table 3:** Comparison of 30‐day, 1‐year and 2‐year outcomes in TAVR patients with and without RASP

	30 days post TAVR				1 year post TAVR				2 years post TAVR			
	Total	No RASP	RASP	P value	Total	No RASP	RASP	P value	Total	No RASP	RASP	P value
No.	598	483	115		598	483	115		598	483	115	
Death [*n* (%)]												
All‐cause death	23 (3.8)	15 (3.1)	8 (7.0)	0.062	83 (13.9)	57 (11.8)	26 (22.6)	0.003	124 (20.7)	85 (17.6)	39 (33.9)	<0.001
CV death	23 (3.8)	15 (3.1)	8 (7.0)	0.062	56 (9.4)	38 (7.9)	18 (15.7)	0.010	74 (12.4)	48 (9.9)	26 (22.6)	<0.001
Neurologic events [*n* (%)]	9 (1.5)	6 (1.2)	3 (2.6)	0.385	12 (2.0)	7 (1.4)	5 (4.3)	0.061	16 (2.7)	10 (2.1)	6 (5.2)	0.098
Ischaemic stroke (*n*)		6	2			7	4			10	5	
Haemorrhagic stroke (*n*)		0	1			0	1			0	1	
Bleeding and transfusions [*n* (%)]	8 (1.3)	6 (1.2)	2 (1.7)	0.677								
Vascular and assess‐related complications [*n* (%)]	11 (1.8)	9 (1.9)	2 (1.7)	1.000								
Minor (*n*)		6	2									
Major (*n*)		3	0									
Procedural or valve‐related complications [*n* (%)]	8 (1.3)	5 (1.0)	3 (2.6)	0.185								
Acute kidney injury [*n* (%)]	28 (4.7)	16 (3.3)	12 (10.4)	0.001								
New onset LBBB [*n* (%)]	53 (8.9)	33 (6.8)	20 (17.4)	<0.001								
New permanent PM [*n* (%)]	34 (5.7)	30 (6.2)	4 (3.5)	0.255	44 (7.4)	36 (7.5)	8 (7.0)	0.854				

CV, cardiovascular; LBBB, left bundle branch block; PM, pacemaker; RASP, relative apical sparing pattern; TAVR, transcatheter aortic valve replacement.

### Prognostic performance of RASP along with other potential risk factors for predicting outcomes post‐TAVR

Multivariable binary logistic regression analysis revealed that RASP (OR = 2.968, 95% CI 1.279–6.890, *P* = 0.011), transapical TAVR approach (OR = 5.283, 95% CI 2.194–12.724, *P* < 0.001) and higher septal E/E′ (>27, OR = 2.599, 95% CI 1.152–5.863, *P* = 0.021) were independently associated with AKI post‐TAVR (*Table* *4*). RASP was the only factor independently associated with new‐onset LBBB post‐TAVR (OR = 2.839, 95% CI 1.540–5.235, *P* < 0.001), irrespective of TAVR approach, BMI, EuroSCORE II, pre‐procedural albumin levels and septal E/E′.

**Table 4:** Prognostic risk factors for predicting 30‐day, 1‐year and 2 year outcomes of TAVR patients

	**Acute kidney injury**		**New onset LBBB**	
	**Adjusted OR (95% CI)**	***P* value**	**Adjusted OR (95% CI)**	***P* value**
Transapical approach	5.283 (2.194–12.724)	<0.001		n.s.
Body mass index ≤25.5 kg/m^2^		n.s		n.s.
EuroSCORE II >6.9%		n.s.		n.s.
Albumin <4.0 g/dL		n.s.		n.s.
Septal E/E′ > 27	2.599 (1.152–5.863)	0.021		n.s.
RASP	2.968 (1.279–6.890)	0.011	2.839 (1.540–5.235)	<0.001

	**1‐year CV death**		**2‐year CV death**	
	**Adjusted HR (95% CI)**	**P value**	**Adjusted HR (95% CI)**	***P* value**
Transapical approach		n.s.		n.s.
BMI mass index ≤25.5 kg/m^2^	1.728 (1.008–2.964)	0.047	1.709 (1.069–2.732)	0.025
EuroSCORE II >6.9%		n.s.		n.s.
Albumin <4.0 g/dL	2.996 (1.758–5.103)	<0.001	2.400 (1.499–3.842)	<0.001
Septal E/E′ > 27		n.s.		n.s.
RASP	1.682 (0.939–3.013)	0.081	2.005 (1.221–3.291)	0.006

All models were adjusted for age and sex. All parameters were added into models with ‘Enter’ method. The median values in the 2‐year CV death group, that is, EuroSCORE II > 6.9%, albumin <4.0 g/dL, and septal E/E′ > 27, were determined as the cutoffs of prognostic parameters.

CI, confidence interval; CV, cardiovascular; E/E′, ratio of early transmitral Doppler flow velocity to early diastolic tissue velocity (septal); HR, hazard ratio; LBBB, left bundle branch block; LVEF, left ventricular ejection fraction; n.s., not significant; OR, odds ratio; RASP, relative apical sparing pattern; TAVR, transcatheter aortic valve replacement.

Regarding CV mortality, multivariable Cox regression analysis showed that low albumin (<4.0 g/dL) was a strong independent predictor of both 1‐year (HR = 2.996, 95% CI 1.758–5.103, *P* < 0.001) and 2‐year (HR = 2.400, 95% CI 1.499–3.842, *P* < 0.001) CV mortality. A BMI ≤ 25.5 kg/m^2^ was also associated with increased risk of CV death at both 1 year (HR = 1.728, 95% CI 1.008–2.964, *P* = 0.047) and 2 years (HR = 1.709, 95% CI 1.069–2.732, *P* = 0.025). RASP showed a significant association with 2‐year CV mortality (HR = 2.005, 95% CI 1.221–3.291, *P* = 0.006), while its association with 1‐year CV mortality did not reach statistical significance (HR = 1.682, *P* = 0.081). These associations remained significant regardless of TAVR approach, EuroSCORE II and septal E/E′.

Subgroup analysis revealed that RASP was significantly associated with higher 2‐year CV mortality after TAVR in patients with preserved LVEF (≥50%, *Table* *5*), and this association remained significant after multivariable adjustment (adjusted HR = 2.154, 95% CI 1.217–3.815, *P* = 0.008). When stratified by GLS, RASP was significantly associated with increased 2‐year cardiovascular mortality in patients with impaired GLS (≤15.5%) (adjusted HR = 1.896, 95% CI 1.062–3.385, *P* = 0.031), but not in those with GLS > 15.5% (adjusted HR = 2.285, 95% CI 0.826–6.319, *P* = 0.111). Although a trend toward increased 1‐year mortality with RASP was observed across most subgroups, these associations did not reach statistical significance after adjustment.

**Table 5:** Subgroup analysis of RASP for predicting CV related outcomes stratified by LVEF and GLS_Avg

	Total	No RASP	RASP	*P* value	Adjusted HR (95% CI)^a^	*P* value
1‐year CV death	**56/598 (9.4)**	**38/483 (7.9)**	**18/115 (15.7)**	**0.010**		
LVEF						
≥50%	42/453 (9.3%)	29/369 (7.9%)	13/84 (15.5%)	0.030	1.626 (0.822–3.216)	0.162
<50%	14/145 (9.7%)	9/114 (7.9%)	5/31 (16.1%)	0.179		
GLS_Avg						
>15.5%	18/232 (7.8%)	16/212 (7.5%)	2/20 (10.0%)	0.659		
≤15.5%	38/366 (10.4%)	22/271 (8.1%)	16/95 (16.8%)	0.016	1.779 (0.911–3.476)	0.092
2‐year CV death	**74/598 (12.4)**	**48/483 (9.9)**	**26/115 (22.6)**	**<0.001**		
LVEF						
≥50%	56/453 (12.4%)	36/369 (9.8%)	20/84 (23.8%)	<0.001	2.154 (1.217–3.815)	0.008
<50%	18/145 (12.4%)	12/114 (10.5%)	6/31 (19.4%)	0.220		
GLS_Avg						
>15.5%	24/232 (10.3%)	19/212 (9.0%)	5/20 (25.0%)	0.041	2.285 (0.826–6.319)	0.111
≤15.5%	50/366 (13.7%)	29/271 (10.7%)	21/95 (22.1%)	0.005	1.896 (1.062–3.385)	0.031

CI, confidence interval; CV, cardiovascular; GLS_Avg, global longitudinal strain averaged by 18 segments; HR, hazard ratio; LVEF, left ventricular ejection fraction; RASP, relative apical sparing pattern; TAVR, transcatheter aortic valve replacement.

^a^ Adjusted for age, sex, TAVR approach, BMI < 25.5 kg/m^2^, EuroSCORE II > 6.9%, albumin <4.0 g/dL, and septal E/E′ > 27 with ‘Enter’ method.

### Risk score‐based stratification of 2‐year CV mortality

**Figure 3:**
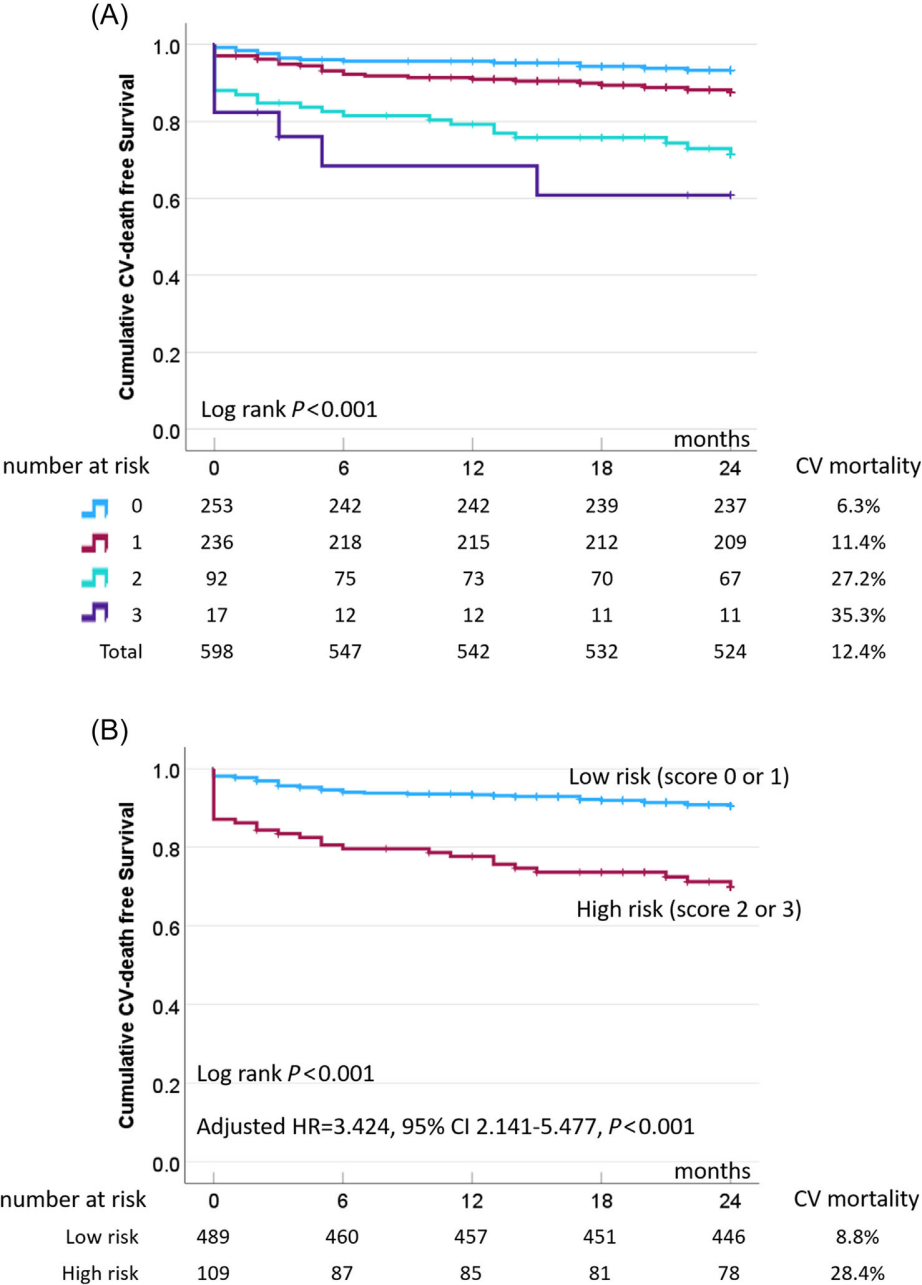
Risk score‐based stratification of 2‐year cardiovascular CV mortality following TAVR. (A) Kaplan–Meier curves illustrating 2‐year CV survival in patients stratified by a composite risk score based on the presence of three independent predictors: relative apical sparing pattern (RASP), low serum albumin (<4.0 g/dL), and low body mass index (BMI ≤ 25.5 kg/m^2^). Each risk factor contributed one point, yielding scores ranging from 0 to 3. Higher risk scores were associated with stepwise increases in 2‐year CV mortality (log‐rank *P* < 0.001). (B) Multivariable Cox regression analysis demonstrating the adjusted HR for 2‐year CV mortality in high‐risk patients (score 2 or 3) compared with those with score 0 or 1. CI, confidence interval; CV, cardiovascular; HR, hazard ratio; RASP, relative apical sparing pattern of left ventricular longitudinal strain; TAVR, transcatheter aortic valve replacement.

To facilitate clinical risk stratification, we developed a simple composite risk score by assigning one point for each of the three independent predictors of 2‐year cardiovascular (CV) mortality identified in the multivariable analysis: presence of RASP, low pre‐procedural serum albumin (<4.0 g/dL) and low BMI (≤25.5 kg/m^2^). This scoring system generated four risk categories (scores 0 to 3). The 2‐year CV mortality rates increased progressively with higher scores: 6.3% (16/253) for score 0, 11.4% (27/236) for score 1, 27.2% (25/92) for score 2 and 35.3% (6/17) for score 3. Kaplan–Meier analysis revealed a clear and statistically significant separation of survival curves across these groups (log‐rank *P* < 0.001; *Figure* *3* *A*). In multivariable Cox regression analysis, patients categorized as high risk (score 2 or 3) exhibited a more than 3‐fold higher risk of 2‐year CV mortality compared with those with lower scores (adjusted HR 3.424, 95% CI 2.141–5.477, *P* < 0.001), independent of other clinical and echocardiographic covariates.

## Discussion

This study introduces a semiquantitative method for identifying the RASP of LS in patients undergoing TAVR and confirms its prognostic value. Compared with the quantitative approach, the semiquantitative method demonstrated stronger predictive power for 2‐year cardiovascular (CV) mortality. We evaluated RASP alongside clinical and echocardiographic parameters and found it independently associated with both post‐procedural complications and long‐term mortality. Key findings include (1) RASP prevalence was 19.2% and associated with AKI, new‐onset LBBB, and increased CV mortality; (2) independent correlates of RASP included diastolic dysfunction and concentric hypertrophy; (3) low serum albumin (<4.0 g/dL) and low BMI (≤25.5 kg/m^2^) were also independent predictors of mortality; and (4) combining these three markers into a simple risk score substantially improved stratification for 2‐year CV mortality.

### RASP prevalence and clinical relevance

The apical sparing pattern of LS was introduced as a key feature of CA, aiding in its differentiation from other causes of LV hypertrophy,^3^ regardless of amyloid type and hypertrophy severity.^17^, ^18^, ^19^ However, its definition varies across studies, leading to inconsistent prevalence estimates.^2^, ^5^, ^20^, ^21^ RASP assessment methods include quantitative, semiquantitative and visual approaches. While quantitative assessment provides high accuracy in distinguishing CA, it is time‐consuming, whereas semiquantitative and visual methods require further validation. Reported RASP prevalence in CA ranges from 26% to 70%, depending on assessment methodology. Data on RASP prevalence in AS patients remain limited, though Saito et al. found semiquantitative and quantitative RASP in 15% and 27% of severe AS patients with preserved LVEF, respectively.^6^ In our study, RASP was relatively common among TAVR patients, with 19.2% exhibiting semiquantitative RASP and 21.4% showing quantitative RASP. Notably, our semi‐quantitative definition—apical LS > 3 × basal LS in at least three LV walls—demonstrated the strongest predictive value for cardiovascular mortality at 2 years post‐TAVR.

TAVR patients with RASP had a distinct clinical profile, including higher EuroSCORE II, lower BMI, reduced albumin levels, and a greater prevalence of atrial fibrillation and hyperuricemia. These characteristics, alongside LV concentric hypertrophy, advanced diastolic dysfunction, and significant MAC, suggest that systemic inflammation, ventricular remodelling and altered haemodynamics contribute to RASP development.^22^, ^23^, ^24^ While previous studies have linked RASP primarily to CA, ^25^, ^26^ emerging evidence—including our findings—suggests that RASP can also occur in severe AS, independent of amyloidosis.^5^, ^10^ Further studies are needed to refine its pathophysiological significance and prognostic implications in TAVR patients.

Our findings differ from those of Fernández et al., who analysed RASP in a broader CA population.^8^ Their study primarily focused on amyloid‐related mechanisms, whereas our analysis highlights RASP in AS patients undergoing TAVR, where additional factors—such as pressure overload, fibrotic remodelling and myocardial energetics—likely play a role. Differences in strain quantification techniques and endpoint definitions may also contribute to the observed discrepancies. Further studies are needed to clarify the prognostic role of RASP in distinct cardiac populations.

### Prognostic values of RASP in TAVR patients

LVEF and GLS are widely used markers of systolic function, but their prognostic value in AS patients with preserved LVEF remains uncertain. LVEF is highly afterload‐dependent and may remain within normal limits despite the presence of myocardial fibrosis and longitudinal dysfunction in advanced AS stages.^27^, ^28^ Similarly, GLS, assessed by speckle‐tracking echocardiography, has demonstrated value for risk stratification in asymptomatic severe AS with preserved LVEF^29^ and has shown predictive power for all‐cause mortality in severe AS, regardless of treatment modality.^30^ In our cohort, neither LVEF nor GLS was associated with outcomes, a finding consistent with emerging evidence suggesting their limited discriminative power in this population.^28^, ^29^ This may be attributed to compensatory hypertrophy preserving LVEF until late‐stage disease^22^ and the averaging nature of GLS, which might potentially diluting regional abnormalities. In contrast, RASP appears more sensitive to strain heterogeneity and fibrosis patterns, particularly at basal segments experiencing the highest wall stress.^23^ Additionally, its prognostic advantage may be attributed to differential myocardial layer responses to afterload^24^ or early amyloid infiltration, though amyloidosis was not systematically assessed in this cohort. These findings suggest that deformation heterogeneity, as captured by RASP, may provide a more refined prognostic marker in AS patients undergoing TAVR.

RASP emerged as an independent predictor of 30‐day, 1‐year and 2‐year CV mortality in TAVR patients, outperforming LVEF and GLS. This finding builds on prior studies highlighting its prognostic relevance, particularly in patients with LV hypertrophy.^9^ Additionally, we found significant associations between RASP and post‐procedural AKI and new‐onset LBBB, both of which are linked to increased mortality risk. ^31^, ^32^ These findings suggest that incorporating RASP into risk assessment could enhance post‐TAVR prognostication.

Our results align with findings by Ferreira et al., who demonstrated that a visual apical‐sparing strain pattern predicts higher all‐cause mortality post‐TAVR.^10^ Notably, RASP remained an independent predictor of CV mortality even after adjusting for pre‐procedural albumin levels—a well‐established prognostic factor. Its prognostic value was particularly evident in patients with preserved global and longitudinal systolic function (LVEF ≥50% or GLS > 12%), supporting the notion that RASP provides additional risk stratification beyond conventional systolic indices in severe AS.^6^

### Pre‐procedural albumin level and BMI associated with outcomes in TAVR patients

Hypoalbuminemia, a marker of malnutrition and systemic inflammation, has been associated with adverse outcomes in both cardiac and non‐cardiac surgical populations.^33^, ^34^, ^35^ Koifman et al. previously demonstrated that low pre‐procedural serum albumin (<3.5 g/dL), combined with BMI, independently predicted mortality after TAVR.^36^ Our findings build on this by showing that both low albumin (<4.0 g/dL) and low BMI (≤25.5 kg/m^2^) are independently associated with increased CV mortality at 1 year and 2 years post‐TAVR. Using ROC analysis, we identified 25.5 kg/m^2^ as the optimal BMI threshold for predicting 2‐year CV mortality. Patients below this cut‐off had significantly higher mortality risk, suggesting that lower BMI—likely reflecting frailty or sarcopenia—is a key prognostic marker in this elderly TAVR population, rather than the absence of obesity per se.

### Risk score‐based stratification

To improve risk stratification in TAVR patients, we developed a composite risk score incorporating three independent predictors of 2‐year CV mortality: RASP, low pre‐procedural serum albumin and low BMI. This simple additive model effectively differentiated mortality risk across four categories, with a stepwise increase in 2‐year CV mortality corresponding to higher scores. Patients with two or more risk factors had a markedly elevated risk—over threefold—compared with those with zero or one. The clear separation of survival curves supports the discriminative power of this score. By combining functional (RASP), nutritional (albumin) and anthropometric (BMI) parameters, this model offers a practical tool for identifying high‐risk individuals who may benefit from intensified monitoring, nutritional optimization or tailored procedural planning prior to TAVR.

### Clinical implications

Although pathological confirmation of CA was not available, our findings highlight that RASP—regardless of its aetiology—functions as an independent prognostic marker in TAVR patients. Beyond its association with post‐procedural complications such as AKI and new‐onset LBBB, RASP also predicts long‐term cardiovascular mortality. These results support the integration of advanced strain imaging into the pre‐TAVR assessment to identify subtle myocardial dysfunction patterns that may otherwise go unrecognized. Importantly, the combination of RASP with low pre‐procedural albumin and low BMI enhances risk stratification and identifies a particularly vulnerable subgroup of patients. This high‐risk phenotype may benefit from intensified peri‐procedural surveillance, nutritional optimization and tailored management strategies aimed at improving clinical outcomes. As such, our study underscores the utility of combining functional, nutritional and structural parameters for individualized care planning in the TAVR population.

## Study limitations

This retrospective study has several inherent limitations. Although the sample size is relatively modest, it provides real‐world insights and generates hypotheses for future research. The absence of systematic screening for cardiac amyloidosis, particularly wild‐type transthyretin amyloidosis in elderly patients, limits our ability to determine whether RASP reflects myocardial fibrosis, amyloid infiltration, or a combination of both. While our study was not designed to distinguish between these mechanisms, this remains an important consideration. Further studies are warranted to elucidate the underlying pathophysiological basis of RASP. Another limitation is the lack of follow‐up strain imaging, which restricts our ability to assess post‐TAVR strain remodelling and determine whether RASP represents reversible myocardial dysfunction or ongoing pathological changes. Although LVEF and GLS were not associated with outcomes in this cohort, this does not diminish their clinical value. Rather, our findings highlight the potential utility of more refined markers, such as RASP, in capturing subclinical myocardial dysfunction in AS patients with preserved EF. Finally, the observed association between lower BMI and adverse outcomes may reflect the obesity paradox, a phenomenon frequently described in cardiovascular research. Because BMI does not distinguish between fat and lean mass, it may serve as a surrogate for sarcopenia or frailty, both of which are established predictors of poor prognosis in elderly patients.^37^, ^38^ However, because body composition was not systematically assessed in our cohort, this remains a hypothesis‐generating observation.

## Conclusions

In patients undergoing TAVR, RASP represents a distinct myocardial phenotype independently associated with adverse short‐ and long‐term outcomes. When combined with hypoalbuminemia and low BMI, it enables more accurate risk stratification for 2‐year CV mortality. These findings support the integration of advanced strain imaging and nutritional markers into routine pre‐TAVR evaluation to guide personalized clinical decision‐making.

## Funding

This study was supported by grants from the Bundesministerium für Bildung und Forschung (BMBF 01EO1504).

## Conflict of Interest

The authors declare that there is no conflict of interest.

## Supporting information


**Table S1.** Baseline clinical and echocardiography characteristics associated with 30‐day and 1‐year cardiovascular (CV) mortality post TAVR. ^a^ Apical‐basal GLS ratio = $\frac{\mathrm{GLS}\_\text{apical}}{\mathrm{GLS}\_\mathrm{mid}+\mathrm{GLS}\_\text{basal}}$; ^b^ Apical‐basal LS ratio of each LV wall = $\frac{\mathrm{LS}\_\text{apical}}{\mathrm{LS}\_\text{basal}}$. Abbreviations: EuroSCORE, European System for Cardiac Operative Risk Evaluation; NYHA, New York Heart Association; PCI, percutaneous coronary intervention; CABG, coronary artery bypass grafting; eGFR, estimated glomerular filtration rate; TAVR, transcatheter aortic valve replacement; LV, left ventricular; LVEF, left ventricular ejection fraction; LVEDD, end‐diastolic left ventricular dimension; IVSd, end‐diastolic wall thickness of the septum; LVPWd, end‐diastolic wall thickness of the left ventricular posterior wall; LVMi, left ventricular mass indexed to body surface area; RWT, relative wall thickness; LAVi, end‐systolic left atrial volume indexed to body surface area; RAA, end‐systolic right atrial area; RVD_mid, end‐diastolic right ventricular mid diameter; TAPSE, tricuspid annular plane systolic excursion; MAPSE, mitral annular plane systolic excursion; sPAP, systolic pulmonary artery pressure; E/E′, ratio of early transmitral Doppler flow velocity to early diastolic tissue velocity (septal); DD, diastolic dysfunction; AVV_max_, maximum transaortic velocity; AVP_mean_, mean transaortic gradient; AVAi, aortic valve area indexed to body surface area; SVi, stroke volume indexed to body surface area; AR, aortic regurgitation; MR, mitral regurgitation; MAC, mitral annular calcification; GLS_Avg, global longitudinal strain averaged by 18 segments; GLS_apical, GLS averaged by 6 apical segments; GLS_mid, GLS averaged by 6 mid segments; GLS_basal, GLS averaged by 6 basal segments; LS_apical, apical longitudinal strain of one LV wall; LS_basal, basal longitudinal strain of one LV wall.
**Table S2.** Univariable and multivariable Cox regression models of echocardiographic parameters for predicting 1‐year and 2‐year CV mortality risk in TAVR patients.
**Table S3.** Diagnostic test evaluation of quantitative and semi‐quantitative definitions of RASP. ^a^ Apical‐basal GLS ratio = $\frac{\mathrm{GLS}\_\text{apical}}{\mathrm{GLS}\_\mathrm{mid}+\mathrm{GLS}\_\text{basal}}$; ^b^ Apical‐basal LS ratio of each LV wall = $\frac{\mathrm{LS}\_\text{apical}}{\mathrm{LS}\_\text{basal}}$. Abbreviations: GLS, global longitudinal strain; LS, longitudinal strain; GLS_apical, GLS averaged by 6 apical segments; GLS_mid, GLS averaged by 6 mid segments; GLS_basal, GLS averaged by 6 basal segments; LS_apical, apical longitudinal strain of one LV wall; LS_basal, basal longitudinal strain of one LV wall.
