# Supplementary material for: Prognostic value of longitudinal strain relative apical sparing in severe aortic stenosis patients undergoing TAVR
Source: ESC Heart Fail. 2025 Jul 27;12(5):3537–51. doi: 10.1002/ehf2.15365 (PMC12450792; doi:10.1002/ehf2.15365)
Supplement: Supplementary file 1 — Table S1. Baseline clinical and echocardiography characteristics associated with 30‐day and 1‐year cardiovascular (CV) mortality post TAVR. a Apical‐basal GLS ratio = GLS_apicalGLS_mid+GLS_basal; b Apical‐basal LS ratio of each LV wall = LS_apicalLS_basal. Abbreviations: EuroSCORE, European System for Cardiac Operative Risk Evaluation; NYHA, New York Heart Association; PCI, percutaneous coronary intervention; CABG, coronary artery bypass grafting; eGFR, estimated glomerular filtration rate; TAVR, transcatheter aortic valve replacement; LV, left ventricular; LVEF, left ventricular ejection fraction; LVEDD, end‐diastolic left ventricular dimension; IVSd, end‐diastolic wall thickness of the septum; LVPWd, end‐diastolic wall thickness of the left ventricular posterior wall; LVMi, left ventricular mass indexed to body surface area; RWT, relative wall thickness; LAVi, end‐systolic left atrial volume indexed to body surface area; RAA, end‐systolic right atrial area; RVD_mid, end‐diastolic right ventricular mid diameter; TAPSE, tricuspid annular plane systolic excursion; MAPSE, mitral annular plane systolic excursion; sPAP, systolic pulmonary artery pressure; E/E′, ratio of early transmitral Doppler flow velocity to early diastolic tissue velocity (septal); DD, diastolic dysfunction; AVVmax, maximum transaortic velocity; AVPmean, mean transaortic gradient; AVAi, aortic valve area indexed to body surface area; SVi, stroke volume indexed to body surface area; AR, aortic regurgitation; MR, mitral regurgitation; MAC, mitral annular calcification; GLS_Avg, global longitudinal strain averaged by 18 segments; GLS_apical, GLS averaged by 6 apical segments; GLS_mid, GLS averaged by 6 mid segments; GLS_basal, GLS averaged by 6 basal segments; LS_apical, apical longitudinal strain of one LV wall; LS_basal, basal longitudinal strain of one LV wall. Table S2. Univariable and multivariable Cox regression models of echocardiographic parameters for predicting 1‐year and 2‐year CV mortality [file EHF2-12-3537-s001.docx]

**Supplementary Tables**

**Table S1 Baseline clinical and echocardiography characteristics associated with 30-day and 1-year cardiovascular (CV) mortality post TAVR**

|  | **Total** | **30 days post TAVR** | | |  | **1 year post TAVR** | | |
| --- | --- | --- | --- | --- | --- | --- | --- | --- |
|  |  | **No CV-death** | **CV-death** | **P value** |  | **No CV-death** | **CV-death** | **P value** |
| **No.** | 598 (100) | 575 (96.2) | 23 (3.8) |  |  | 542 (90.6) | 56 (9.4) |  |
| **Age (years)** | 81.7±5.7 | 81.7±5.7 | 81.9±5.6 | 0.865 |  | 81.7±5.7 | 81.7±6.1 | 0.941 |
| **Male [n (%)]** | 291 (48.7) | 282 (49.0) | 9 (39.1) | 0.351 |  | 263 (48.5) | 28 (50.0) | 0.833 |
| **Body mass index (kg/m²)** | 27.1±4.5 | 27.2±4.5 | 24.6±5.7 | 0.045 |  | 27.2±4.5 | 26.2±5.3 | 0.139 |
| **≤25.5 kg/m² (%)** | 227 (38.0) | 212 (36.9) | 15 (65.2) | 0.006 |  | 197 (36.3) | 30 (53.6) | 0.011 |
| **Systolic blood pressure (mmHg)** | 135.6±24.3 | 135.8±24.2 | 130.1±27.2 | 0.344 |  | 136.2±24.5 | 130.2±22.0 | 0.111 |
| **Diastolic blood pressure (mmHg)** | 70.6±13.7 | 70.7±13.8 | 69.2±14.0 | 0.676 |  | 70.6±13.8 | 71.0±13.8 | 0.819 |
| **EuroSCORE II (%)** | 5.0 (3.2-8.7) | 5.0 (3.2-8.7) | 6.0 (4.0-10.0) | 0.308 |  | 5.0 (3.1-8.3) | 6.9 (4.5-10.7) | 0.008 |
| **>6.9%** | 217 (36.3) | 206 (35.8) | 11 (47.8) | 0.241 |  | 189 (34.9) | 28 (50.0) | 0.025 |
| **NYHA class II/III/IV (%)** | 15.1/73.4/9.5 | 15.3/73.4/9.4 | 8.7/73.9/13.0 | 0.658 |  | 15.1/73.8/9.0 | 14.3/69.6/14.3 | 0.654 |
| **Comorbidities (%)** |  |  |  |  |  |  |  |  |
| **Obesity** | 22.9 | 23.1 | 17.4 | 0.521 |  | 23.1 | 21.4 | 0.782 |
| **Atrial fibrillation** | 41.6 | 41.4 | 47.8 | 0.539 |  | 40.4 | 53.6 | 0.057 |
| **Hypertension** | 81.4 | 81.7 | 73.9 | 0.344 |  | 81.2 | 83.9 | 0.615 |
| **Diabetes** | 33.8 | 33.6 | 39.1 | 0.580 |  | 33.8 | 33.9 | 0.980 |
| **Dyslipidemia** | 64.0 | 64.3 | 56.5 | 0.443 |  | 64.4 | 60.7 | 0.585 |
| **Hyperuricemia** | 10.2 | 9.7 | 21.7 | 0.074 |  | 10.0 | 12.5 | 0.550 |
| **Coronary artery disease** | 56.4 | 56.3 | 56.5 | 0.987 |  | 56.1 | 58.9 | 0.683 |
| **Previous myocardial infarction** | 13.2 | 13.4 | 8.7 | 0.514 |  | 13.3 | 12.5 | 0.869 |
| **Previous PCI** | 30.3 | 29.9 | 39.1 | 0.345 |  | 29.9 | 33.9 | 0.531 |
| **Previous CABG** | 12.2 | 12.5 | 4.3 | 0.341 |  | 12.2 | 12.5 | 0.944 |
| **Stroke or transient ischemic attack** | 17.1 | 16.9 | 21.7 | 0.570 |  | 16.8 | 19.6 | 0.589 |
| **Peripheral vascular disease** | 11.9 | 11.7 | 17.4 | 0.338 |  | 11.1 | 19.6 | 0.059 |
| **Chronic respiratory diseases** | 23.1 | 22.8 | 30.4 | 0.393 |  | 22.7 | 26.8 | 0.489 |
| **Chronic renal dysfunction** | 58.9 | 58.3 | 73.9 | 0.135 |  | 58.3 | 64.3 | 0.386 |
| **Permanent pacemaker** | 9.7 | 9.7 | 8.7 | 1.000 |  | 9.6 | 10.7 | 0.787 |
| **Biochemical parameters** |  |  |  |  |  |  |  |  |
| **Creatinine (mg/dl)** | 1.19 (0.95-1.50) | 1.19 (0.95-1.48) | 1.34 (0.95-2.20) | 0.112 |  | 1.18 (0.95-1.47) | 1.32 (0.96-1.69) | 0.072 |
| **eGFR (ml/min/1.73qm)** | 55.5 (41.0-70.0) | 56.0 (41.0-71.0) | 45.0 (26.0-60.0) | 0.052 |  | 56.0 (41.0-70.0) | 46.5 (41.0-70.0) | 0.100 |
| **Hemoglobin (g/dl)** | 12.3 (11.2-13.3) | 12.4 (11.2-13.4) | 11.5 (10.4-12.3) | 0.038 |  | 12.4 (11.2-13.4) | 11.6 (10.5-12.6) | 0.001 |
| **Albumin (g/dl)** | 4.2 (4.0-4.4) | 4.2 (4.0-4.5) | 3.8 (3.6-4.3) | <0.001 |  | 4.3 (4.1-4.5) | 4.1 (3.7-4.3) | <0.001 |
| **TAVR approach** |  |  |  | 0.307 |  |  |  | 0.066 |
| **Transfemoral** | 397 (66.4) | 384 (66.8) | 13 (56.5) |  |  | 366 (67.5) | 31 (55.4) |  |
| **Transapical** | 201 (33.6) | 191 (33.2) | 10 (43.5) |  |  | 176 (32.5) | 25 (44.6) |  |
| **Standard echocardiography measurements** |  |  |  |  |  |  |  |  |
| **LVEF (%)** | 57.0±13.0 | 57.0±13.0 | 57.7±12.3 | 0.787 |  | 57.0±13.2 | 57.0±11.3 | 0.971 |
| **≥50%** | 453 (75.8) | 436 (75.8) | 17 (73.9) | 0.834 |  | 411 (75.8) | 42 (75.0) | 0.890 |
| **<50%** | 145 (24.2) | 139 (24.2) | 6 (26.1) |  |  | 131 (24.2) | 14 (25.0) |  |
| **LVEDD (mm)** | 47.9±7.6 | 58.0±7.7 | 46.3±6.9 | 0.309 |  | 48.0±7.7 | 46.6±7.0 | 0.192 |
| **IVSd (mm)** | 11.7±2.0 | 11.7±2.0 | 11.9±2.2 | 0.677 |  | 11.8±2.0 | 11.6±2.1 | 0.603 |
| **LVPWd (mm)** | 11.2±1.9 | 11.2±1.9 | 11.4±2.2 | 0.694 |  | 11.2±1.8 | 10.9±2.1 | 0.228 |
| **LVMi (g/m²)** | 114.4±32.2 | 114.3±32.1 | 115.8±34.1 | 0.830 |  | 114.9±32.1 | 108.8±32.4 | 0.175 |
| **RWT** | 0.48±0.13 | 0.48±0.13 | 0.51±0.16 | 0.372 |  | 0.48±0.13 | 0.48±0.14 | 0.925 |
| **LV concentric hypertrophy (%)** | 37.0 | 36.9 | 39.1 | 0.826 |  | 37.6 | 30.4 | 0.283 |
| **LAVi (ml/m²)** | 44.8±18.9 | 44.6±18.8 | 50.3±19.7 | 0.159 |  | 44.5±18.8 | 47.8±19.1 | 0.215 |
| **RAA (cm²)** | 17.9±6.3 | 17.9±6.2 | 19.9±7.1 | 0.134 |  | 17.7±6.1 | 19.8±7.3 | 0.020 |
| **RVD_mid (mm)** | 27.8±6.7 | 27.9±6.7 | 26.7±6.5 | 0.389 |  | 27.8±6.6 | 27.9±7.0 | 0.939 |
| **TAPSE (mm)** | 18.5±4.9 | 18.5±4.9 | 16.6±4.2 | 0.066 |  | 18.6±4.9 | 17.2±4.5 | 0.050 |
| **MAPSE_septal (mm)** | 7.9±2.1 | 8.0±2.1 | 7.2±1.8 | 0.076 |  | 8.0±2.1 | 7.4±1.7 | 0.058 |
| **MAPSE_lateral (mm)** | 9.3±2.3 | 9.3±2.3 | 8.8±2.3 | 0.283 |  | 9.3±2.4 | 9.0±2.1 | 0.349 |
| **sPAP (mmHg)** | 41.1±13.6 | 40.7±13.3 | 48.8±17.4 | 0.005 |  | 40.5±13.2 | 46.0±15.9 | 0.005 |
| **Septal E/E´** | 23.4±10.6 | 23.0±10.3 | 31.7±13.5 | 0.001 |  | 22.9±10.3 | 27.8±12.7 | 0.005 |
| **DD grade (%)** |  |  |  | 0.078 |  |  |  | 0.321 |
| **Mild** | 37.5 | 38.1 | 21.7 |  |  | 38.4 | 28.6 |  |
| **Moderate** | 50.0 | 49.9 | 52.2 |  |  | 49.4 | 55.4 |  |
| **Severe** | 12.5 | 12.0 | 26.1* |  |  | 12.2 | 16.1 |  |
| **AVV_max_ (m/s)** | 4.2±0.7 | 4.2±0.7 | 4.2±0.7 | 0.828 |  | 4.2±0.7 | 4.1±0.7 | 0.504 |
| **AVP_mean_ (mmHg)** | 46.9±16.2 | 46.9±16.2 | 47.0±16.3 | 0.969 |  | 47.2±16.4 | 44.3±14.2 | 0.215 |
| **AVAi (cm²/m²)** | 0.44±0.11 | 0.44±0.11 | 0.42±0.10 | 0.513 |  | 0.44±0.11 | 0.43±0.10 | 0.690 |
| **SVi (ml/m²)** | 43.5±10.5 | 43.6±10.4 | 42.0±11.0 | 0.486 |  | 43.7±10.4 | 41.6±10.5 | 0.149 |
| **Moderate to severe AR (%)** | 14.0 | 14.1 | 13.0 | 1.000 |  | 14.6 | 8.9 | 0.247 |
| **Moderate to severe MR (%)** | 22.7 | 22.1 | 39.1 | 0.056 |  | 23.2 | 17.9 | 0.360 |
| **Moderate to severe MAC (%)** | 13.2 | 13.0 | 17.4 | 0.529 |  | 12.9 | 16.1 | 0.507 |
| **2D speckle tracking derived longitudinal strain** |  |  |  |  |  |  |  |  |
| **18-segment GLS_Avg** | -14.1±3.9 | -14.2±3.9 | -13.4±3.3 | 0.372 |  | -14.2±3.9 | -13.9±3.6 | 0.619 |
| **Absolute value >12%** | 413 (69.1) | 397 (69.0) | 16 (69.6) | 0.958 |  | 374 (69.0) | 39 (69.6) | 0.922 |
| **Absolute value ≤12%** | 185 (30.9) | 178 (31.0) | 7 (30.4) |  |  | 168 (31.0) | 17 (30.4) |  |
| **GLS_apical** | -19.1±6.1 | -19.2±6.2 | -18.4±5.1 | 0.568 |  | -19.1±6.2 | -19.3±5.5 | 0.795 |
| **GLS_mid** | -13.0±3.8 | -13.0±3.9 | -12.6±3.3 | 0.601 |  | -13.0±3.9 | -12.8±3.6 | 0.651 |
| **GLS_basal** | -10.3±3.4 | -10.3±3.4 | -9.6±3.5 | 0.313 |  | -10.4±3.4 | -9.8±3.6 | 0.217 |
| **Apical-basal GLS ratio ^a^** | 0.85±0.25 | 0.84±0.25 | 0.88±0.32 | 0.451 |  | 0.84±0.25 | 0.90±0.28 | 0.084 |
| **RASP definition** |  |  |  |  |  |  |  |  |
| **If defined as apical-basal GLS ratio ^a^ >1** | 128 (21.4) | 123 (21.4) | 5 (21.7) | 1.000 |  | 113 (20.8) | 15 (26.8) | 0.302 |
| **If defined as ≥3 out of 6 LV walls with apical-basal LS ratio ^b^ >3.0** | 115 (19.2) | 107 (18.6) | 8 (34.8) | 0.062 |  | 97 (17.9) | 18 (32.1) | 0.010 |

^a^ Apical-basal GLS ratio = $\frac{GLS\_apical}{GLS\_mid+GLS\_basal}$; ^b^ Apical-basal LS ratio of each LV wall =$\frac{LS\_apical}{LS\_basal}$

Abbreviations: EuroSCORE, European System for Cardiac Operative Risk Evaluation; NYHA, New York Heart Association; PCI, percutaneous coronary intervention; CABG, coronary artery bypass grafting; eGFR, estimated glomerular filtration rate; TAVR, transcatheter aortic valve replacement; LV, left ventricular; LVEF, left ventricular ejection fraction; LVEDD, end-diastolic left ventricular dimension; IVSd, end-diastolic wall thickness of the septum; LVPWd, end-diastolic wall thickness of the left ventricular posterior wall; LVMi, left ventricular mass indexed to body surface area; RWT, relative wall thickness; LAVi, end-systolic left atrial volume indexed to body surface area; RAA, end-systolic right atrial area; RVD_mid, end-diastolic right ventricular mid diameter; TAPSE, tricuspid annular plane systolic excursion; MAPSE, mitral annular plane systolic excursion; sPAP, systolic pulmonary artery pressure; E/E´, ratio of early transmitral Doppler flow velocity to early diastolic tissue velocity (septal); DD, diastolic dysfunction; AVV_max_, maximum transaortic velocity; AVP_mean_, mean transaortic gradient; AVAi, aortic valve area indexed to body surface area; SVi, stroke volume indexed to body surface area; AR, aortic regurgitation; MR, mitral regurgitation; MAC, mitral annular calcification; GLS_Avg, global longitudinal strain averaged by 18 segments; GLS_apical, GLS averaged by 6 apical segments; GLS_mid, GLS averaged by 6 mid segments; GLS_basal, GLS averaged by 6 basal segments; LS_apical, apical longitudinal strain of one LV wall; LS_basal, basal longitudinal strain of one LV wall.

**Table S2 Univariable and multivariable Cox regression models of echocardiographic parameters for predicting 1-year and 2-year CV mortality risk in TAVR patients**

|  | **1-year CV mortality post TAVR** |  | **Multivariable models** |  |
| --- | --- | --- | --- | --- |
|  | **Crude HR (95% CI)** | **P value** | **Adjusted HR (95% CI)** | **P value** |
| **Body mass index ≤25.5 kg/m²** | 1.973 (1.167-3.337) | 0.011 | 1.975 (1.151-3.390) | 0.014 |
| **Albumin (g/dl)** | 0.279 (0.157-0.495) | <0.001 | 0.364 (0.191-0.693) | 0.002 |
| **Hemoglobin (g/dl)** | 0.804 (0.691-0.935) | 0.005 | 0.873 (0.735-1.039) | 0.126 |
| **RAA (cm²)** | 1.043 (1.006-1.081) | 0.022 | 1.038 (0.999-1.079) | 0.059 |
| **Septal E/E´** | 1.035 (1.014-1.056) | <0.001 | 1.037 (1.013-1.061) | 0.002 |
| **sPAP (mmHg)** | 1.026 (1.008-1.045) | 0.005 | 1.007 (0.987-1.027) | 0.509 |
|  |  |  |  |  |
|  | **2-year CV mortality post TAVR** |  | **Multivariable models** |  |
|  | **Crude HR (95% CI)** | **P value** | **Adjusted HR (95% CI)** | **P value** |
| **Body mass index ≤25.5 kg/m²** | 1.965 (1.245-3.103) | 0.004 | 1.894 (1.177-3.049) | 0.009 |
| **Albumin (g/dl)** | 0.311 (0.187-0.517) | <0.001 | 0.414 (0.232-0.739) | 0.003 |
| **Hemoglobin (g/dl)** | 0.815 (0.714-0.931) | 0.002 | 0.875 (0.751-1.019) | 0.086 |
| **LAVi (ml/m²)** | 1.011 (1.001-1.022) | 0.029 | 0.999 (0.986-1.013) | 0.924 |
| **RAA (cm²)** | 1.036 (1.003-1.070) | 0.031 | 1.026 (0.987-1.067) | 0.197 |
| **TAPSE (mm)** | 0.948 (0.903-0.994) | 0.027 | 0.979 (0.928-1.033) | 0.430 |
| **MAPSE_septal (mm)** | 0.887 (0.791-0.995) | 0.041 | 0.985 (0.864-1.124) | 0.826 |
| **Septal E/E´** | 1.032 (1.014-1.051) | <0.001 | 1.030 (1.009-1.052) | 0.005 |
| **sPAP (mmHg)** | 1.025 (1.009-1.042) | 0.002 | 1.008 (0.991-1.026) | 0.363 |

**Table S3 Diagnostic test evaluation of quantitative and semi-quantitative definitions of RASP**

|  | **Sensitivity (%)** | **Specificity (%)** | **Positive predictive value (%)** | **Negative predictive value (%)** |
| --- | --- | --- | --- | --- |
| **2-year all-cause death** |  |  |  |  |
| **Apical-basal GLS ratio** ^a^ **>1.0** | 29.03 | 80.59 | 28.13 | 81.28 |
| **≥3 walls with apical-basal LS ratio** ^b^ **>3.0** | 31.45 | 83.97 | 33.91 | 82.40 |
| **≥4 walls with apical-basal LS ratio** ^b^ **>3.0** | 16.94 | 93.25 | 39.62 | 81.10 |
| **≥4 walls with apical-basal LS ratio** ^b^ **>2.5** | 25.00 | 85.86 | 31.63 | 81.40 |
|  |  |  |  |  |
| **2-year CV death** |  |  |  |  |
| **Apical-basal GLS ratio** ^a^ **>1.0** | 26.76 | 79.32 | 14.84 | 88.94 |
| **≥3 walls with apical-basal LS ratio** ^b^ **>3.0** | 35.21 | 82.92 | 21.74 | 90.48 |
| **≥4 walls with apical-basal LS ratio** ^b^ **>3.0** | 16.90 | 92.22 | 22.64 | 89.17 |
| **≥4 walls with apical-basal LS ratio** ^b^ **>2.5** | 25.35 | 84.82 | 18.37 | 89.40 |

^a^ Apical-basal GLS ratio = $\frac{GLS\_apical}{GLS\_mid+GLS\_basal}$; ^b^ Apical-basal LS ratio of each LV wall =$\frac{LS\_apical}{LS\_basal}$

Abbreviations: GLS, global longitudinal strain; LS, longitudinal strain; GLS_apical, GLS averaged by 6 apical segments; GLS_mid, GLS averaged by 6 mid segments; GLS_basal, GLS averaged by 6 basal segments; LS_apical, apical longitudinal strain of one LV wall; LS_basal, basal longitudinal strain of one LV wall.
